# Enhancement of Antinociceptive Effect by Co-administration of Amitriptyline and *Crocus Sativus* in a Rat Model of Neuropathic Pain

**Published:** 2017

**Authors:** Bahareh Amin, Samira Hosseini, Hossein Hosseinzadeh

**Affiliations:** a *Cellular and Molecular Research Center,Department of Physiology and Pharmacology, Faculty of Medicine, Sabzevar University of Medical Sciences, Sabzever, Iran. *; b *Department of Pharmacodynamics and Toxicology, School of Pharmacy, Mashhad University of Medical Sciences, Mashhad, Iran. *; c *Pharmaceutical Research Center, Department of Pharmacodynamics and Toxicology, School of Pharmacy, Mashhad University of Medical Sciences, Mashhad, Iran.*

**Keywords:** Amitriptyline, Saffron, Neuropathic pain, Antinociceptive, Allodynia, Combination therapy

## Abstract

The aim of this study was to evaluate the anti-nociceptive effects of a low, sub-effective dose of amitriptyline, in combination with the different doses of ethanolic and aqueous extracts of Crocus sativus following sciatic nerve chronic constriction injury (CCI) in rats.

Amitriptyline (3, 10 and 30 mg/Kg, i.p.) and the extracts (25, 50 and 100 mg/Kg, i.p.), were separately administered at the time of CCI for 7 consecutive days. In combination therapy, the sub-antinociceptive dose of amitriptyline (3 mg/Kg) was given with the three different doses of extracts for seven days. Mechanical allodynia, thermal allodynia and thermal hyperalgesia were evaluated by von Frey, acetone and radiant heat tests, respectively, 1 day before and on days 3, 5 and 7 after surgery.

Co-administration of amitriptyline (3 mg/Kg) with aqueous extract (50, 100 mg/Kg,) produced more potent cold anti-allodynic (P < 0.01 and P < 0.001, respectively) as well as thermal anti-hyperalgesic (P < 0.05) effects than that produced by each of them. Amitriptyline (3 mg/Kg) plus ethanolic extract (50, 100 mg/Kg) produced more potent cold anti-allodynic (P < 0.05 and P < 0.001, respectively) as well as thermal anti-hyperalgesic (P < 0.05) effects as compared with the sum effects produced by each of them. Mechanical anti-allodynia effect was only potentiated with the co-administration of amitriptyline with the high dose of aqueous extract (100 mg/Kg, P < 0.001).

Our study supports the use of saffron as an adjunctive to amitriptyline to improve the therapeutic outcome in the management of neuropathic pain.

## Introduction

Neuropathic pain is defined as a chronic pain syndrome following a lesion or dysfunction of the normal sensory pathways either in the peripheral or central nervous system and is clinically characterized by spontaneous pain (ongoing, paroxysms) as well as evoked types of pain: hyperalgesia (an increased response to painful stimuli) and allodynia (pain perception to stimuli that do not normally evoke pain) (1). Chronic pain is difficult to treat and responds poorly to current approved drugs. Tricyclic antidepressants such as amitriptyline are the mainly used drugs in the management of this chronic disease (2). Unfortunately, their anti-cholinergic, anti-histaminergic and anti-adrenergic properties contribute to a poor side-effect profile and limited patient tolerability. As a result, these drugs are primarily used as adjuncts to other treatments (3, 4)**. **Meanwhile, discrepancies exist on the effects of the amitriptyline in different models of neuropathic pain. De Vry *et al*. (2004), reported that amitriptyline (32 mg/Kg) was active against thermal hyperalgesia, but had no mechanical anti-allodynia effect up to 128 mg/Kg (5). In another study, thermal allodynia was attenuated with the low dose of 10 mg/Kg of intraperitoneal amitriptyline; while, mechanical allodynia was unaffected up to 30 mg/Kg (6). In a model of neuropathic pain on rats, acute or chronic administration of amitriptyline inhibited thermal hyperalgesia but not mechanical allodynia (7, 8). In the tibial nerve injury model, mechanical allodynia was partially reversed by amitriptyline (32 mg/Kg, i.p.) (9). Suzuki *et al. *(2012), reported that acute administration of amitriptyline (60 mg/Kg, p.o.) had little effect on cold and mechanical allodynia, and its effects were not statistically significant in CCI rats (10). 

For years, medicinal plants are believed to be an important source of new drugs (11). Saffron, a spice comprised of the dried stigmas of *Crocus sativus *L., (family: Iridaceae), is now used worldwide for coloring and flavoring food preparations (12). In traditional folklore, it was believed to be useful in the treating numerous illnesses including cough, asthma, menstruation problems, insomnia, pain, colic, chronic uterine hemorrhage, cardiovascular disorders and tumors (12, 13). In various pharmacological studies, saffron extracts and its active constituents, safranal and crocin, have shown anti-cancer (14), anti-oxidant (15), anti-convulsant (16), anti-depressant (17, 18), anti-anxiety (19), memory improvement properties (20), anti-nociceptive and anti-inflammatory properties (21). 

Previously, it was reported that ethanolic and aqueous extracts of *Crocus sativus* (*C. sativus*) attenuated pain behaviors in a model of neuropathic pain (22). This study was aimed to investigate therapeutic potential of a sub-effective dose of amitriptyline with different sub-effective or less effective doses of ethanolic and aqueous extracts of saffron in rats underwent chronic constriction injury (CCI)-induced neuropathic pain to see whether this combination have potentiation effects or not. 

## Methods and Materials

*Animals*

Adult male Wistar rats weighing 200–250 g at the time of surgery were employed in the present study. Animals were randomly selected from the animal center of School of Pharmacy, Mashhad University of Medical Sciences, Iran and were maintained under standard environmental conditions (12–12-h light/dark cycle at 22 °C), with free access to food pellets and water. Experimental protocol was approved by Mashhad University of Medical Sciences and conformed to Internationally Accepted Principles for Laboratory Animal Use and Care (23).

*Materials*

The stigmas of *C. sativus* L. collected from Ghaen (Khorasan province, northeast of Iran), was purchased from Novin Saffron Co. (Mashhad, Iran) and analyzed in accordance to ISO/TS 67321–2. Aqueous and ethanolic extracts were extracted from saffron in our laboratory. Amitriptyline was a gift from Daroupakhsh Pharmaceutical Co. (Tehran, Iran). The saffron extracts and amitriptyline were dissolved in normal saline (0.9%) immediately before injection and administered intraperitoneally, once a day for 7 consecutive days. Ketamine and xylazine were purchased from Alfasan Co., (Woerden, Netherlands).

*Preparation of extracts*

Ethanolic extract: 100 g of saffron powder was mixed with 1 L ethanol 80% and left on a shaking incubator at 8 °C for 72 h. The solution was then filtered and dried on 40 ºC water bath and stored in -4 °C and away from light.

Aqueous extract: Saffron powder was mixed with distilled water (1:50 W/V) and left on a shaking incubator at 8 °C for 48 h. The solution was centrifuged at 4000 g for 10 min. The resulting supernatant was retained and sediment was suspended in the half amount of mentioned distilled water and placed on the shaking incubator for another 24 h. The centrifugation was repeated again and yielded supernatant was separated, stored at −20 °C and subsequently freeze dried (22). 

*Induction of neuropathic pain*

Animals were anesthetized with a cocktail of ketamine and xylazine (60/4 mg/Kg). To prepare the unilateral CCI, The method described by Bennet and Xie was generally followed. The left sciatic nerve was exposed at the mid-thigh level by blunt dissection just proximal to its trifurcation. About 12 mm of nerve was freed of adhering tissue and four chromic catgut ligatures (4-0) were tied loosely around it with about 1 mm spacing (24).

*Study protocol*

At the time of experiment, rats were divided into eighteen groups of six each. These groups comprised of: Group 1: Rats subjected to CCI and received normal saline (NS). Group 2: Sham-operated rats underwent a similar surgical procedure, except for the nerve ligation. Groups 3-5: CCI rats treated with different doses (25, 50, or 100 mg/Kg) of ethanolic extract. Groups 6-8: CCI rats treated with different doses (25, 50, or 100 mg/Kg) of aqueous extract. Groups 9-11: CCI rats treated with different doses (3, 10, or 30 mg/Kg) of amitriptyline. The dosage selection in single administration of drugs was based on previous studies (6, 22) and in combination therapies was according to the results obtained from individual administration of drugs. Groups 12-14: CCI rats treated with 3 mg/Kg of amitriptyline and different doses (25, 50, or 100 mg/Kg) of ethanolic extract in combination. Groups 15-17: CCI rats treated with 3 mg/Kg of amitriptyline and different doses (25, 50, or 100 mg/Kg) of aqueous extract in combination. 

The pain related behaviors induced following CCI have been established to peak within a week after injury. Hence a time course of 7-day was performed (5). All drugs were administered by intraperitoneal rout, immediately after surgery and continued daily thereafter until day 7.

*Behavioral measurements*

Behavioral assessments were made before (day 0) and at specific times, days 3, 5 and 7, after surgery. All tests were performed between 8:00 AM and 1:00 PM.

*Von Frey test for mechanical allodynia*

The von Frey filaments test was performed within the sciatic innervation area of the hind paws as described previously to assess mechanical allodynia (24). Briefly, calibrated monofilaments (Stoelting, Wood Dale, IL, USA) were applied randomly to the left hind paws to elicit paw withdrawal responses. The smallest monofilament that evoked paw-withdrawal responses on three of five trials was taken as the mechanical withdrawal threshold. Nociceptive responses for mechanical sensitivity were expressed as mechanical paw withdrawal threshold (PWT) in grams. The cut off pressure was 60 gram (g). 

*Acetone test for cold allodynia*

Cold sensitivity was quantified by measuring the frequency of paw withdrawal in response to non-noxious cold chemical stimuli, acetone (25). Acetone drop was applied 5 times to the hind paw, with a gap of 5 min between the tests. Nociceptive responses for cold sensitivity were expressed as the paw withdrawal frequency (PWF). Percent inhibition (PI) of drugs to innocuous stimulus acetone was evaluated as following equation:

[(AUC of vehicle)-(AUC of post-compound) /AUC of vehicle]×100

*Radiant heat test for thermal hyperalgesia*

In order to evaluate the thermal threshold, withdrawal latencies were measured with a plantar test unit (model 7370, UgoBasile, Italy). A source of infrared light (radiant heat) was directed towards the hind paw plantar surface and the latency for paw withdrawal from the radiant heat was automatically determined by a photoelectric cell detecting the reflected light from the paw. A cut-off time was set at 30 sec (s) to avoid tissue damage. Nociceptive responses for thermal sensitivity were expressed as the mean of three or four paw withdrawal latencies (PWL) in Sec (26). The overall anti-nociceptive effects of drugs during the 7-day observation period were expressed as the AUC of the maximum possible effect (%MPE) according to the following equation:

 [(AUC _Latency time compound_) – (AUC _Latency time vehicle_) / (AUC _Latency time sham_ − AUC _Latency time vehicle_)] ×100) (27).

*Statistical analysis*

A two-way analysis of variance (ANOVA) with repeated measures followed by Bonferroni’s post-hoc analysis was used to examine the time-courses of behavioral changes after various treatments. Kruskal–Wallis ANOVA, followed by the Mann–Whitney U test was used to measure variance between groups on a given testing day. The whole anti-nociceptive effects elicited by drugs during the 7-day observation period were expressed as the area under the curve (AUC) calculated by the trapezoidal method. If the observed AUC of combination therapy exceeded the sum of AUCs of individual drugs, it was considered to show potentiation and if they were similar, it was considered to show an additive anti-nociceptive effect. The observed AUC of combination therapy was considered to show sub-additive interaction, whether it was lower to the theoretical sum (28). The AUC values for drug combinations were compared with the expected value using Student›s t-test. 

## Results

*Effect of C. sativus extracts on the mechanical allodynia*

**Figure 1:**
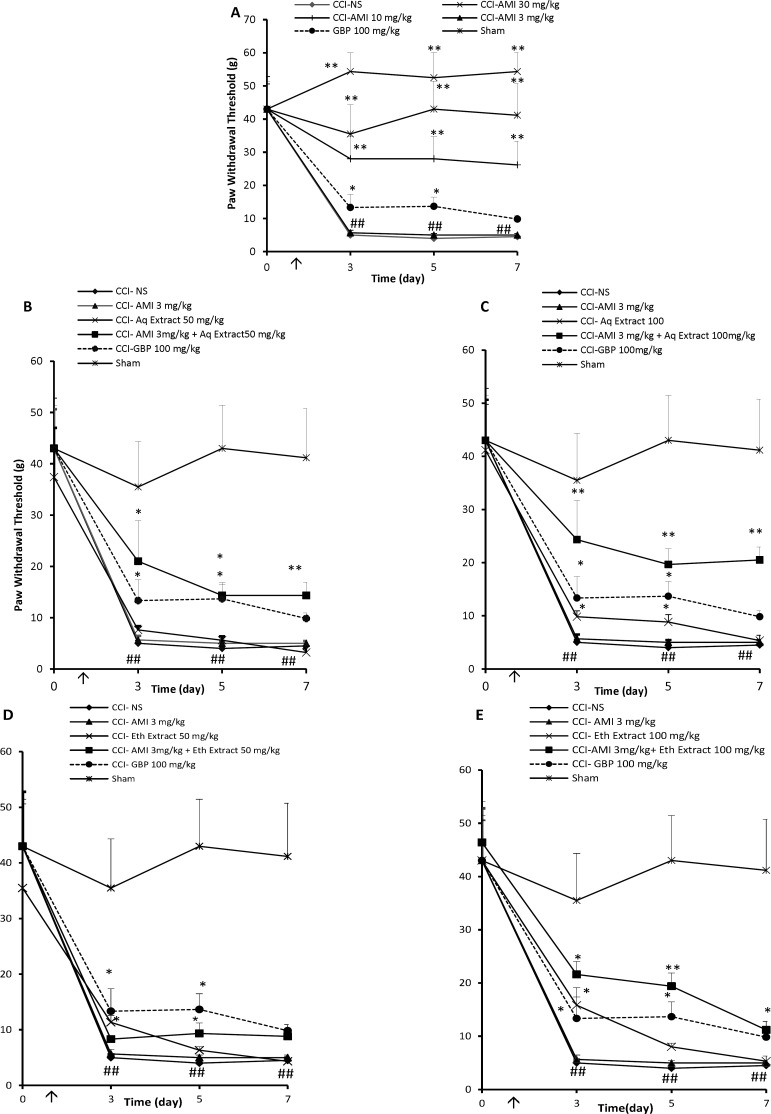
Mechanical anti-allodynia effects (time course) of daily administration of A: amitriptyline alone (AMI, 3, 10 and 30 mg/Kg), B: AMI 3 mg/kg plus saffron aqueous (Aq) extract (50 mg/Kg), C: AMI 3 mg/Kg plus saffron aqueous (Aq) extract (100 mg/Kg), D: AMI 3 mg/Kg plus saffron ethanolic (Eth) extract (50mg/Kg), and E: AMI 3 mg/Kg + saffron ethanolic (Eth) extract (100 mg/Kg), on the CCI-induced mechanical allodynia in rats. Administration of drugs started intraperitoneally on the day of surgery and continued until day 7 post-surgery. The upward arrow indicates the time of CCI and starting the drug administration. Von Frey tests were conducted one day before surgery (Day 0) as well as at 3, 5 and 7 days after that. Data are presented as mean±SEM of six determinants. Two-way analysis of variance (ANOVA) with repeated measures followed by Bonferroni’s post-hoc analysis was used to examine the time-courses of behavioral changes after various treatments *P<0.05, **P<0.01 vs control group (CCI + NS). ##P < 0.01 control (CCI + NS) group vs. sham group

CCI rats treated with NS exhibited significant mechanical allodynia as compared to sham-operated animals which increased progressively during the study (Figure 1A, P < 0.01). Amitriptyline at the doses of 10 and 30 mg/Kg significantly improved response to von Frey stimulation (Figure 1A, P < 0.01). Administration of low dose of amitriptyline (3 mg/Kg) for 7 consecutive days was not superior to NS in reducing mechanical hypersensitivity (Figure 1A). The aqueous extracts of saffron at the dose of 25 did not alter von Frey responses in CCI animals (data not shown). Aqueous extract, 50 mg/Kg, for 7 consecutive days did not alter von Frey responses in CCI animals (Figure 1B), while 100 mg/Kg of extract produced a transient anti-nociceptive on days 3 and 5 (Figure 1 C, P < 0.05). 

Amitriptyline, 3 mg/Kg, combined with the 25 mg/Kg aqueous extract showed no anti-allodynic effect with respect to control group (Table 1). Although the combination of amitriptyline + aqueous extract (3 + 50 mg/Kg) yielded an AUC higher than that produced by control group (107 ± 23 and 49.5 ± 5.3, respectively; P < 0.05, Figure1 B), it was not significantly different from the expected AUC resulting from the sum of the individual values (Table 1). Time course of tactile sensitivity changes of animals after treatment with combination of amitriptyline + aqueous extract (3 + 100 mg/Kg), once daily for 7 days can be seen in Figure 1C. The antinociception produced by such combination represented a potentiated effect obtained with 129.1 ± 13 area units (au), while respective value obtained with the sum of individual agents was 119 ± 5.6 a.u. (P < 0.001 by Student›s t test, Table 1). Some increase in the PWT of animals treated with amitriptyline + ethanolic extract was noted; however, these changes did not reach a statistical significance even with the highest dose of this extract (Figure 1D and E). 

**Figure 2:**
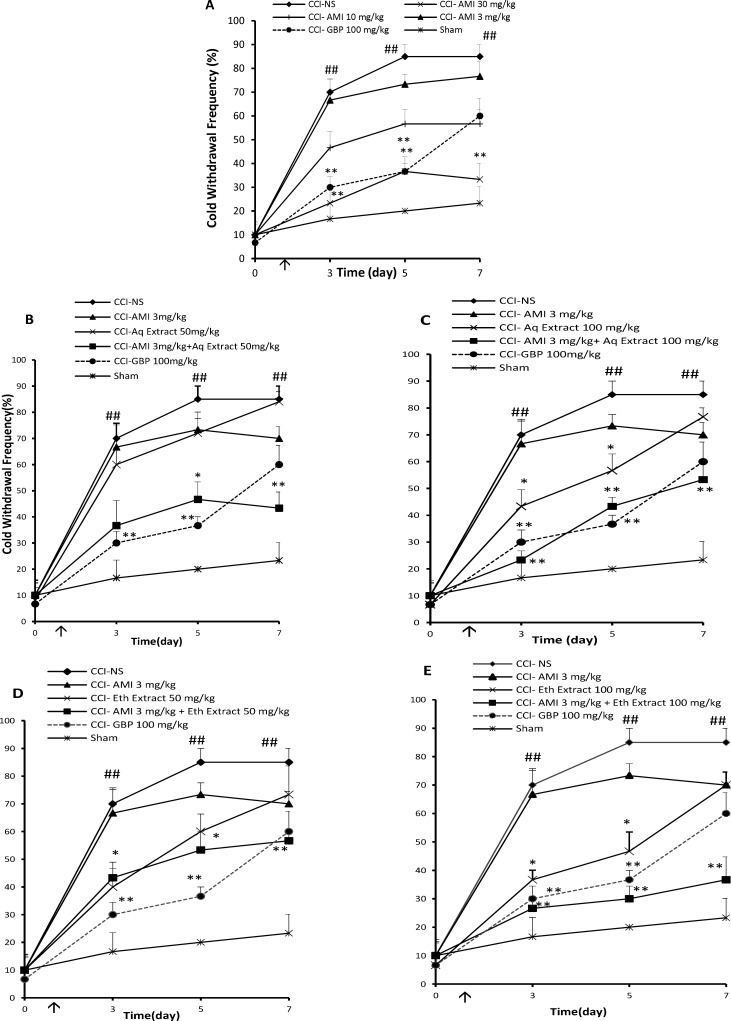
Cold antiallodynia effects (time course) of daily administration of A: amitriptyline alone (AMI, 3, 10 and 30 mg/Kg), B: AMI 3 mg/Kg plus saffron aqueous (Aq) extract (50 mg/Kg), C: AMI 3 mg/Kg plus saffron aqueous (Aq) extract (100 mg/Kg), D: AMI 3 mg/Kg plus saffron ethanolic (Eth) extract (50mg/Kg), and E: AMI 3 mg/Kg + saffron ethanolic (Eth) extract (100 mg/Kg), on the CCI- induced cold allodynia in rats. Administration of drugs started intraperitoneally on the day of surgery and continued until day 7 post-surgery. The upward arrow indicates the time of CCI and starting the drug administration. Acetone tests were conducted one day before surgery (Day 0) as well as at 3, 5 and 7 days after that. Data are presented as mean±SEM of six determinants. Two-way analysis of variance (ANOVA) with repeated measures followed by Bonferroni’s post-hoc analysis was used to examine the time-courses of behavioral changes after various treatments *P<0.05, **P<0.01 *vs* control group (CCI + NS). ##P < 0.01 control (CCI + NS) group vs. sham group

**Figure 3:**
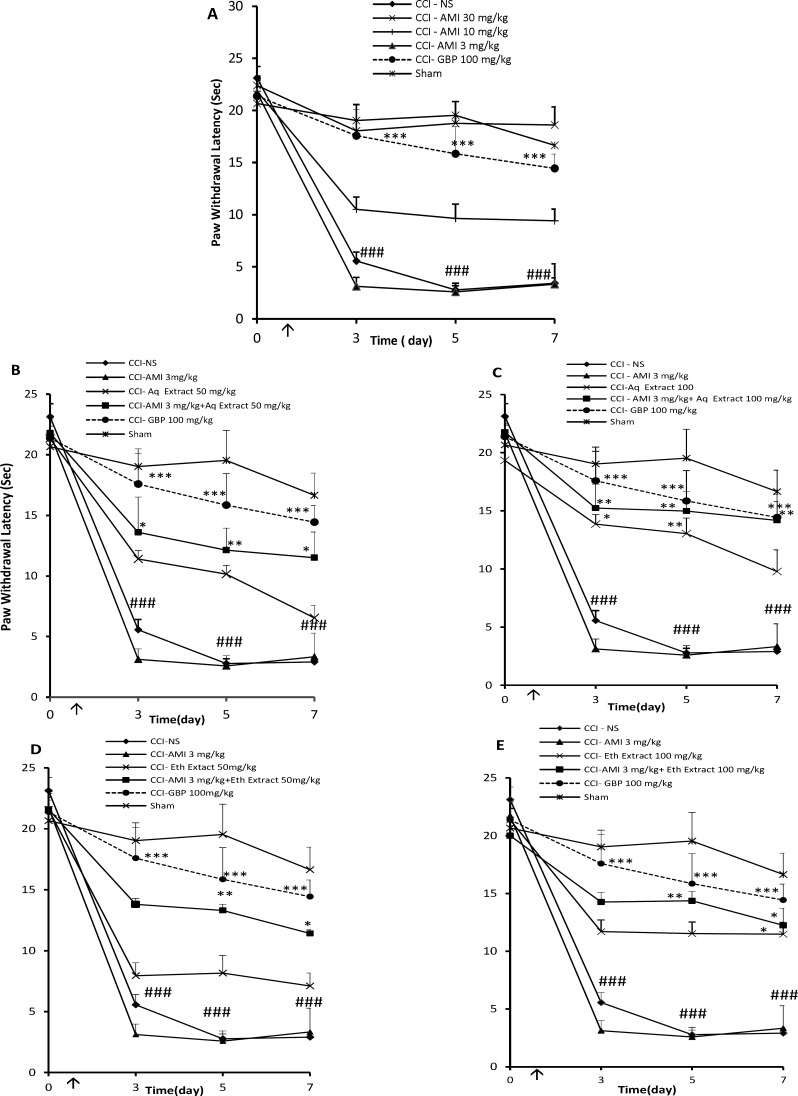
Thermal antihyperalgesia effects (time course) of daily administration of A: amitriptyline alone (AMI, 3, 10 and 30 mg/Kg), B: AMI 3 mg/Kg plus saffron aqueous (Aq) extract (50 mg/Kg), C: AMI 3 mg/Kg plus saffron aqueous (Aq) extract (100 mg/Kg), D: AMI 3 mg/Kg plus saffron ethanolic (Eth) extract (50mg/Kg), and E: AMI 3 mg/Kg + saffron ethanolic (Eth) extract (100 mg/Kg), on the CCI- induced thermal hyperalgesia in rats. Administration of drugs started intraperitoneally on the day of surgery and continued until day 7 post-surgery. The upward arrow indicates the time of CCI and starting the drug administration. Radiant heat tests were conducted one day before surgery (Day 0) as well as at 3, 5 and 7 days after that. Data are presented as mean±SEM of six determinants. Two-way analysis of variance (ANOVA) with repeated measures followed by Bonferroni’s post-hoc analysis was used to examine the time-courses of behavioral changes after various treatments *P<0.05, **P<0.01 *vs* control group (CCI + NS). ###P < 0.001 control (CCI + NS) group vs. sham group

**Table 1:** The AUC of seven days administration of amitriptyline and aqueous extract of *C. sativus* individually and in combination, as well as their interaction consequence on the behavioral characterizations in CCI model of neuropathic pain. Comparison among groups was performed by two-way analysis of variance (ANOVA) with repeated measures followed by Bonferroni’s post-hoc test. For analysis of AUCs, student's t-test was used. * P < 0.05, ** P < 0.01, *** P < 0.001 vs. control group; # P< 0.05, ## P < 0.01, ### P < 0.001 observed effect vs. expected effect combinations

**Treatment** **(mg/kg, i.p.)**	**AUC of mechanical anti-allodynia effects**	**Results of combination therapy Vs. single administration of drugs**	**AUC of** **percent inhibition** **of cold allodynia**	**Results of combination therapy Vs. single administration of drugs**	**AUC of** **maximum possible effect against thermal hyperalgesia**	**Results of combination therapy Vs. single administration of drugs**
Ami 3	53.1 ± 5		9.8 ± 5.2		7.3±2.2	
Aqueous Extract 25	51.2 ±5.2		3 ± 2.9		0.9 ± 1.5	
Observed effect of Ami 3+Aq extract 25	57 ± 5.5	Sub-additive	1.6 ± 3.6	Sub-additive	4.3 ± 1.7	Sub-additive
Expected effect of Ami 3+Aq extract 25	104.3 ± 3.43		12.8 ± 3.16		8.2 ± 1.8	
Aqueous extract 50	52 ± 7.2		12 ± 4.6		39.5±2.5	
Observed effect of Ami 3+Aq extract 50	107 ± 23*	Sub-additive	46 ± 8.1**, ##	Potentiated	58.4±11.2*, #	Potentiated
Expected effect of Ami 3+Aq extract 50	105.1 ± 3.9		21.8 ± 3.08		46.8±3.07	
Aqueous extract 100	66 ± 9.7^**, ###^		29 ± 3.5**		57.1 ± 8.2*	
Observed effect of Ami 3+Aq extract 100	129.1 ± 13	Potentiated	51 ± 4.03***, ###	Potentiated	74.7 ± 10.9**	Potentiated
Expected effect of Ami 3+Aq extract 100	119.1 ± 5.6		38.8 ± 3.4		67.8±5.8	
Control group (CCI+NS)	49.5 ± 5.3		0		0	

**Table 2:** The AUC of seven days administration of amitriptyline and ethanolic extract of *C. sativus* individually and in combination, as well as their interaction consequence on behavioral characterizations in CCI model of neuropathic pain. Comparison among groups was performed by two-way analysis of variance (ANOVA) with repeated measures followed by Bonferroni’s post-hoc test. For analysis of AUCs, student's t-test was used. * P< 0.05, ** P < 0.01, *** P < 0.001 vs. control group; # P< 0.05, ### P < 0.001 observed effect vs. expected effect combinations

Treatment (mg/kg, i.p.)	AUC of mechanical anti allodynia effects	Results of combination therapy Vs. single administration of drugs	AUC of percent inhibition of cold allodynia	Results of combination therapy Vs. single administration of drugs	AUC of Maximum possible effect of thermal hyperalgesia	Results of combination therapy Vs. single administration of drugs
Ami 3	53.1 ± 5		9.8 ± 5.2		7.3±2.2	
Eth extract 25	52.4 ± 5.4		3 ± 4.5		0.4 ± 1.3	
Observed effect of Ami 3+Eth extract 25	62.1 ± 3.5	Sub-additive	3.3 ± 3.5	Sub-additive	2.87 ± 0.02	Sub-additive
Expected effect of Ami 3+Eth extract 25	105.5 ± 7.1		12.8 ± 3.51		7.7 ± 1.8	
Eth extract 50	58.5 ± 5.8		24 ± 4.8		28.3±4	
Observed effect of Ami 3+Eth extract 50	70.3 ± 5.2	Sub-additive	36 ± 4.5*,#	Potentiated	56±9.7*,#	Potentiated
Expected effect of Ami 3+Eth extract 50	111.6 ± 6.1		33.8 ± 4.4		35.6±2.2	
Eth extract 100	70 ± 11		39.5 ± 4.4***, ###		51.5±6.03	
Observed effect of Ami 3+Eth extract 100	116.9 ± 7.4*	Sub-additive	60.9 ± 5.2	Potentiated	65.2 ± 10.2**, #	Potentiated
Expected effect of Ami 3+Eth extract 100	123.1±12.4		49.3±4.8		62.8±4.6	
Control group (CCI+NS)	49.5 ± 5.3		0		0	

*Effect of C. sativus extracts on the cold allodynia*

The overall anti-allodynic effect against acetone stimulation was expressed as the AUC of PWF during the study. Baseline innocuous cold stimulus (acetone drop) testing, performed on the day before surgery (day 0), revealed no differences among the groups in response to acetone drop. Three days after the surgery, behavior tests showed that CCI rats treated with normal saline exhibited significantly higher paw withdrawal frequencies than sham operated rats, indicative of cold allodynia (Figure 2A, P < 0.01). The paw withdrawal responses to cold stimulation in sham rats remained unchanged throughout the study. Administration of amitriptyline only at the dose of 30 mg/Kg, i.p., once daily for 7 days) delayed the development of cold allodynia in CCI rats as compared to NS-treated CCI rats (Figure 2A, P < 0.01). The combination of sub-effective doses of aqueous or ethanolic extracts + amitriptyline (25 mg/Kg + 3 mg/Kg) for 7 days yielded AUCs, which were lower than the expected AUCs resulting from the sum of the individual values of drugs (Tables 1 and 2). 

When administered alone, aqueous and ethanolic extracts (50 mg/Kg, i.p.) for 7 days had no significant effect on cold sensitivity (12 ± 4.6 and 24 ± 4.8, respectively). However, treatment with the extracts and sub effective dose of amitriptyline (3 mg/Kg, i.p.) delayed the development of cold allodynia (Figure 2B and D respectively). The analysis of the AUCs also showed a potentiation effect (P < 0.01 and P < 0.05 respectively by Student›s t test, Tables 1 and 2). In combination, 100 mg/Kg of aqueous and ethanolic extracts for 7 days, which transiently attenuated paw withdrawal responses to cold stimulation, strongly potentiated the analgesic effect of amitriptyline (Figure 2C and 2E), .i.e., the area under the curve observed with the combinations were significantly higher than the one corresponding to the theoretical sum of individual effects (P < 0.001 by Student›s t test, Tables 1 and 2). The area under the curve presented by gabapentin showed significant cold anti allodynia effect (50.4 ± 2.4 a.u., P < 0.001).

*Effect of C. sativus extracts on the thermal hyperalgesia*

Baseline Hargreaves testing, performed on the day before surgery (day 0), revealed no differences among the groups in this test. After 3 days of CCI, rats treated with normal saline exhibited significantly shorter paw latencies than sham animals, which is an indicative of thermal hyperalgesia (P < 0.01, Figure 3A). The administration of amitriptyline only at the dose of 30 mg/Kg, i.p., once daily for 7 days, resulted in a significant change in the reaction latency compared to NS- treated CCI rats in the radiant heat test (P < 0.001, Figure 3A). The combination of sub-effective doses of aqueous or ethanolic extracts + amitriptyline (25 mg/Kg + 3 mg/Kg) for 7 days did not show anti-hyperalgesia effect (Tables 1 and 2). 

When administered alone, aqueous and ethanolic extracts (50 mg/Kg, i.p.), for 7 days had no significant effect against the radiant heat stimulus. However, co-administration of amitriptyline and extracts (3 mg/Kg + 50 mg/Kg) induced an increase in the reaction latency time compared to vehicle treated animals or each of them alone (P < 0.05 by Student›s t test, Figure 3B, 3D and Tables 1, 2 respectively).

Seven days drug treatment with amitriptyline (3 mg/Kg) and aqueous extract (100 mg/Kg) significantly elevated the threshold of the pain evoked by radiant heat. The analysis of the AUC from the hyperalgesia test also shows that extracts potentiated amitriptyline analgesic effect (P < 0.05 by Student›s t test, Figure 3C and Table 1). 

Although 100 mg/Kg of ethanolic extract suppressed thermal hyperalgesia associated with CCI, its effect was disappeared during the study. As indicated in Figure 3E, co-administration of ethanolic extract plus amitriptyline (100 mg/Kg + 3 mg/Kg) significantly prolonged the mean latency of the response to heating. The AUC observed with this combination was significantly higher than the theoretical sum of individual effects (65.2 ± 10.2 vs. 62.8 ± 4.6 a.u, respectively; P < 0.05; Student›s t test). 

## Discussion and Conclusion

*Discussion*

Amitriptyline, a tricyclic antidepressant, exhibits anti-nociceptive effects in the preclinical models of neuropathic pain and is widely used as an analgesic for chronic pain therapy (2, 29).

In the present study, amitriptyline relieved mechanical allodynia from the dose of 10 mg/Kg, i.p. Similarly, amitriptyline (10 mg/Kg, i.p., bid) attenuated mechanical allodynia in neuropathic rats (30). 

Cold allodynia and thermal hyperalgesia were only attenuated with the high dose of 30 mg/Kg, which is consistent with the Berrocoso *et al. *study, that the sub-chronic administration of 10 mg/Kg amitriptyline attenuated mechanical allodynia but not thermal allodynia (31).

Analyses of the AUCs of the whole thermal anti-hyperalgesic or cold anti-allodynic effects of sub-effective dose of amitriptyline with less effective doses of *C. sativus* extracts showed more antinociceptive effects, when compared with the sum effects expected from alone administration of each of them. It should be noted that only the application of aqueous extract and amitriptyline (100 + 3 mg/Kg, respectively) was able to potentiate the decreased tactile sensitivity of CCI rats. As constituents of aqueous extract are more water hydrophilic/soluble ingredients such as crocins, with less permeability to CNS and ingredients of ethanolic extract are more oil soluble/hydrophobic including crocetin and safranal, with better permeability to CNS, more involvement of peripheral mechanisms might be hypothesized in the induction of mechanical allodynia rather than thermal hyperalgesia or cold allodynia. 

However, neuroprotective effects of crocins have been reported in many studies (32). As a result, another possible explanation is that crocins which are exist in much more amounts in the aqueous extract may be able to penetrate to the blood-brain barrier and responsible for anti-allodynic effects observed with the aqueous extract. Intraperitoneal administration of crocin protected against spatial memory deficit induced by chronic cerebral hypoperfusion in rats (33). In an ischemia-reperfusion brain model, the infarct volume was reduced by the intravenous injection of crocin (34). After oral administration, crocin converts to crocetin (35). It has been demonstrated that crocetin crosses the blood–brain barrier when saffron extract is administered intraperitoneally (36). However, more investigations are required to clarify such difference. 

Although not reported in our study, sedation was observed with the higher dose range of amitriptyline especially at the dose of 30 mg/Kg, whereas combination therapies used in this study showed no observed sedative effect, indicating more reliable results from the behavioral tests in the combination of drugs. In combination therapy, application of lower doses of each drug is accompanied with fewer or milder adverse effects and also greater analgesic efficacy (37). The potentiation effect obtained with the combinations of amitriptyline and ethanolic/aqueous extracts of saffron is likely to be mediated through recruitment of different mechanisms. The role of monoamine neurotransmitters, serotonin/5-hydroxytriptamine (5-HT) and norepinephrine, via descending inhibitory pathways has been demonstrated in the modulation of pain. Selective serotonin reuptake inhibitors (SSRIs) are becoming increasingly administered in the treatment of chronic neuropathic pain (38).

Increased level of serotonin and norepinephrine are thought to participate to the analgesic activity of amitriptyline in the central synapses of the pain system (39). Through antidepressant studies of saffron, it was suggested that crocin constituent may act via the uptake inhibition of dopamine and norepinephrine, while safranal might act through the reuptake of serotonin (17). 

Increased contents of glutamate and aspartate, major excitatory neurotransmitters, have been implicated in the pathogenesis of neuropathic pain (40). We previously reported that safranal caused a significant decrease in the concentration of glutamate and aspartate in the extracellular space of hippocampus following systemic administration of kainic acid in anesthetized rats (12). Saffron extracts and crocetin were demonstrated to bind the phencyclidine (PCP) binding side of the N-methyl-D aspartate (NMDA) receptor and the sigma (1) receptor in brain, while the crocins and picrocrocin were not effective (41). In a recent study by Mao and Yang, amitriptyline alleviated the mechanical allodynia in neuropathic animals through up-regulating excitatory amino acid transporters (EAATs) (30). EAAT2 or glutamate transporter 1 (GLT-1) is a predominantly astrocytic transporter that is responsible for about 90% of glutamate uptake in the brain. GLT-1 is widely recognized by neuroscientists as a promising target to manage CNS diseases caused by excessive glutamate transmission such as chronic pain. Consequently, decrease in the glutamate concentration by the extracts and amitriptyline might be at least one of the involved mechanisms in the potentiating the anti-nociceptive effects elicited by our combination therapy. 

Gama-aminobutyric acid (GABA) release and GABA synthesizing enzyme glutamic acid decarboxylase decrease following chronic constriction injury of the sciatic nerve (42). Amitriptyline downregulates spinal cord GABA_B _receptor expression (43). Safranal, via activation of benzodiazepine binding cites of GABA_A _receptor complex, displayed an antiabsence seizure activity in rats (44). Amitriptyline also inhibits adenosine uptake; interacts with opioid mechanisms and blocks neuronal Ca2+ and Na+ channels (2). It has been reported that the opioid receptors may not be involved in the analgesic action of crocin (45). Oxidative stress is one of the important determinants in the pathogenesis of neuropathic pain (46, 47). Antioxidant and anti-inflammatory activities of saffron extracts and their bioactive constituents including crocin, crocetin and safranal demonstrated in various studies (47-49) which may have a role in its anti-allodynic, anti-hyperalgesic and augmenting antinociceptive efficacy of amitriptyline. It is however need to determine the precise mechanisms involved in the potentiating antinociceptive of amitriptyline with saffron’s extracts. 

Depression is one of concerning co-morbidities in neuropathic pain, which reduces considerably the quality of life of patients (50). Although we did not evaluate depressive behavior in CCI rats however, antidepressant effects of saffron have been proved in our previous studies (17, 51). In addition, saffron improved fluoxetine induced sexual dysfunction in two randomized double-blind placebo-controlled study (52, 53).

With respect to the fact that analgesic effects of antidepressants is independent of their antidepressive action, and occurs at the lower doses (54), combining saffron’s extract with amitriptyline might improve depressive behaviors as well as pain behaviors in chronic constriction injury animals.

In conclusion, as applying sub effective dose of amitryptiline in combination with extracts at doses of 50 and 100 mg/kg showed more antinociceptive effects. Morover, data from clinical trials have shown that saffron is well tolerated (55). Our study support the use of *C. sativus* extracts especially aqueous extract as an adjunctive therapy with amitriptyline in patients suffering from neuropathic pain.
